# Advanced Radiology Utilization in a Tertiary Care Emergency Department from 2001 to 2010

**DOI:** 10.1371/journal.pone.0112650

**Published:** 2014-11-18

**Authors:** Shin Ahn, Won Young Kim, Kyung Soo Lim, Seung Mok Ryoo, Chang Hwan Sohn, Dong Woo Seo, Myoung Kwan Kwak, Jae Chol Yoon

**Affiliations:** 1 Department of Emergency Medicine, University of Ulsan College of Medicine, Asan Medical Center, Seoul, Korea; 2 Department of Emergency Medicine, Seoul Medical Center, Seoul, Korea; 3 Department of Emergency Medicine, Chonbuk National University Hospital, Jeonju, Korea; Erasmus Medical Centre, Netherlands

## Abstract

**Objective:**

To evaluate the utilization trends of advanced radiology, i.e. computed tomography (CT) and magnetic resonance imaging (MRI), examination in an emergency department (ED) of an academic medical center from 2001 to 2010.

**Patients and Methods:**

We assessed the overall CT and MRI utilization, and the ED patient encounters. Each examination was evaluated according to the patient’s age and anatomically relevant regions.

**Results:**

During the study period, 737,760 patient visited the ED, and 156,287 CT and 35,018 MRI examinations were performed. The number of annual ED patients increased from 63,770 in 2001 to 94,609 in 2010 (P = 0.018). The rate of CT utilization increased from 105.5 per 1000 patient visits in 2001 to 289.2 in 2010 (P<0.001), and the rate of MRI utilization increased from 8.1 per 1000 patient visits in 2001 to 74.6 in 2010 (P<0.001). In all of the patient age groups, the overall CT and MRI utilization increased. The greater the patient age, the more likely the use of advanced radiology [CT: 87.1 per 1000 patients in age <20 vs. 293.9 per 1000 in age>60 (P<0.001); MRI: 5.1 per 1000 patients in age <20 vs. 108.7 per 1000 in age>60 (P<0.001)]. Abdomen-pelvis (40.2%) and the head (35.7%) comprised the majority of CT scans, while the head (86.4%) comprised the majority of MRI examinations. The rates of advanced radiology use increased across all anatomical regions, with the highest increase being in chest CT (5.9 per 1000 to 49.2) and head MRI (7.2 per 1000 to 61.9).

**Conclusion:**

We report a three-fold and nine-fold increase in the use of CT and MRI, respectively, during the study period. Additional studies will be required to understand the causes of this change and to determine the effect of advanced radiology utilization on the patient outcome.

## Introduction

Computed tomography (CT) use has steadily increased during recent years and has become a vital patient-evaluation component in the emergency department (ED) [1]–[5]. This undoubtedly reflects the increased availability and the improved diagnostic capability of CT scanners in various clinical settings. However, the increased use of CT scans has contributed to the increase in health care expenditures, ionizing radiation exposure, and the length of stay in the ED [6]. To date, little is known regarding how CT use has changed in Korea and whether these changes have been extensive or limited to certain types of imaging studies. Despite its limited availability and the time-consuming nature, some reports have shown that the use of magnetic resonance imaging (MRI) was also increased in the ED [6], [7]. However, the extent of this increase and its relationship to the type of imaging study for which it is used has not been fully investigated.

In this study, we attempt to obtain a better understanding of the utilization pattern of advanced radiology technologies, including CT scanning and MRI, in a tertiary care ED in Korea. We attempted to identify the types of studies with the largest increases and which might suggest areas of potential need for resident training and education for interpreting commonly performed advanced radiology examinations.

## Materials and Methods

The study was performed in a tertiary–care, academic medical center with a recent annual ED census of approximately 104,000 patients. We performed a retrospective, electronic chart review of all patients who underwent CT or MRI scanning during their stay in the ED from 2001 to 2010.

Data regarding patient demographics and the total number of patient encounters per year were recorded. The number of radiology examinations completed was categorized according to the image modality, i.e. CT or MRI, the year, and the types of imaging studies according to the anatomical regions. These anatomical regions included the head, neck, chest, abdomen-pelvis, face, spine, and extremities. However, due to the relatively small numbers of patients examined in some anatomical regions, the neck, face, spine, and extremities were grouped into other groups in CT scans, and for MRI, the study types were divided into head and others. When a patient had more than two sites scanned at a single time, the radiology utilization rate was calculated separately according to the anatomical regions. Patient age was classified into four categories, i.e. <20, 20–40, 40–60, and >60 years.

The primary objective of this study was to determine whether trends in the usage of advanced radiology examinations are seen in both modalities, i.e. CT and MRI, regardless of the patient age group. As a secondary analysis, we also attempted to determine whether any different study types, according to anatomical regions, have most significantly contributed to the annual changes in the advanced radiology utilization rate.

To analyze the trends in ED imaging utilization, radiographic imaging per 1,000 ED visits was calculated. The utilization pattern of both CT and MRI scanning were evaluated and analyzed with standard descriptive statistics using Microsoft Excel 2007 (Microsoft, Redmond, WA, USA) and linear regression analysis with R software version 3.1.0 (R Foundation for Statistical Computing, Vienna, Austria. URL http://www.R-project.org/.).

As our data were obtained retrospectively, and patients were managed without any intent to perform research, this study qualified for exemption from institutional review board review of Asan Medical Center. Informed consent was waived, and patient information was anonymized prior to its analysis.

## Results

All patient visits between January 1, 2001 and December 31, 2010 were included in our study, and a total of 737,760 ED patient visits were reviewed for analysis. During the same period, 156,287 (21.2%) total CT examinations and 35,018 (4.8%) total MRI examinations were performed during patient stays in the ED. There were 386,415 (52.4%) male patients, and male patients were more likely to undergo CT scanning (male 22.8% vs. female 19.4%, P<0.001). However, a gender difference was not found in the MRI scans (male 4.8%, vs. female 4.7%, P = 0.128) (Table 1).

**Table 1:** Demographic data and overall advanced radiology examination utilization rate.

Characteristics	Number (%)	CT utilization	MRI utilization
Total (n, %)	737760 (100)	156287 (21.2)	35018 (4.8)
Gender (n, %)			
Male	386415 (52.4)	88256 (22.8)	18481 (4.8)
Female	351345 (47.6)	68031 (19.4)	16537 (4.7)
Patient age (years)			
<20	201698 (27.3)	17567 (87.1)*	1032 (5.4)*
20–40	161125 (21.8)	32696 (202.9)*	2947 (18.3)*
40–60	194857 (26.4)	53109 (272.6)*	11472 (58.9)*
>60	180053 (24.4)	52913 (293.9)*	19566 (108.7)*
Missing or unknown	27 (0.0)		

*Calculated per 1000 ED visits.

CT = computed tomography; MRI = magnetic resonance imaging.

**Figure 1:**
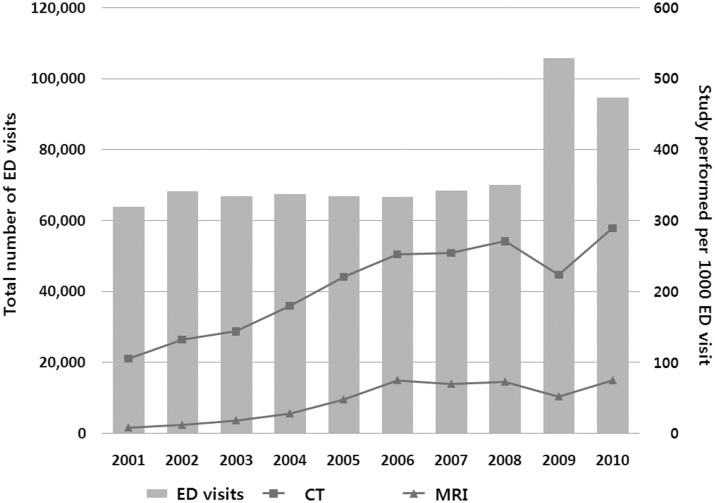
Number of ED visits and advanced radiology utilization by year. ED = Emergency Department; CT = computed tomography; MRI = magnetic resonance imaging.

The number of annual ED patient visits increased from 63,770 in 2001 to 94,609 in 2010 (48.4% increase, P = 0.018), however, the trend was not significant from the year 2001 to 2008 (P = 0.051). In 2009, the outbreak of influenza H1N1 took place and the number of annual visits had increased from 69,997 to 105,640. The total number of advanced radiology examinations increased throughout the 10-year period, i.e. the annual number of CTs increased 406% (6,730 to 27,360) and MRI increased 1,360% (519 to 7,060). After adjusting for annual ED patient visits, overall CT utilization per 1000 ED visits also increased significantly from 105.5 per 1000 ED visits in 2001 to 289.2 per 1000 in 2010 (ß = 19.3, P<0.001). Overall MRI utilization also increased significantly from 8.1 per 1000 ED visits in 2001 to 74.6 per 1000 in 2010 (ß = 7.9, P<0.001) (Figure 1).

**Figure 2:**
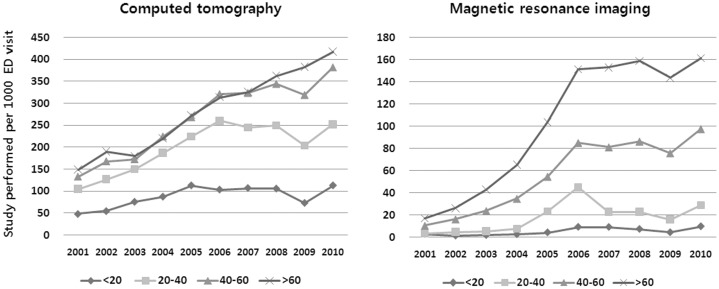
Age groups and changes of studies performed by year. ED = Emergency Department.

When patient age was classified into four categories, i.e. <20, 20–40, 40–60, and >60 years, there were 27.3%, 21.8%, 26.4%, and 24.4% patients in each group. The older the patient age group, the more likely the use of advanced radiology examinations (CT: 87.1 per 1000 ED visits in age <20; 202.9 per 1000 in age 20–40; 272.6 per 1000 in age 40–60; 293.9 per 1000 in age>60, P<0.001; and MRI: 5.4 per 1000 ED visits in age <20; 18.3 per 1000 in age 20–40; 58.9 per 1000 in age 40–60; and 108.7 per 1000 in age>60, P<0.001). During each year of the study period, advanced radiology use in the ED was greater in older patients than in younger patients, and the overall advanced radiology utilization rate according to the age group also increased throughout the 10-year period. However, this increase was not evenly distributed. The slopes of increase in CT utilization rate were higher in older age groups: <20 years (ß = 5.4, P = 0.027); 20–40 years (ß = 15.5, P = 0.002); 40–60 years (ß = 27.4, P<0.001); and >60 years (ß = 30.5, P<0.001). In 2001, 149.0 CT scans per 1000 ED visits were done in >60 years patients, and increased to 417.7 per 1000 in 2010, The overall MRI utilization increase was also significant in all age groups except <20 years: <20 years (ß = 0.8, P = 0.010); 20–40 years (ß = 2.8, P = 0.044); 40–60 years (ß = 10.2, P<0.001); and >60 years (ß = 18.3, P<0.001). In 2001, 16.9 MRI scans per 1000 ED visits were done in >60 years patients and increased to 161.2 per 1000 in 2010 (Figure 2).

**Figure 3:**
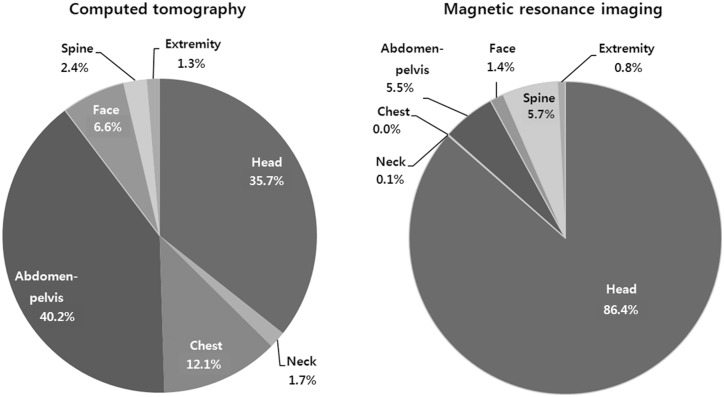
Advanced radiology examinations in anatomical regions.

CT examination of the abdomen-pelvis, head, and chest comprised the majority of the CT scans, i.e. 40.2%, 35.7%, and 12.0% respectively. MRI examinations of the head comprised 86.4% of the total number of MRI examinations, and MRI of the spine comprised 5.7% of the examinations (Figure 3). The percentages of increase of CT and MRI scans were greater than the increase in the ED patient volume each year (Table 2).

**Table 2:** Number of advanced Radiology and Emergency Department visits 2001–2010.

Year	CT	% change	MRI	% change	ED visits	% change
2001	6729	NA	518	NA	63770	NA
2002	8987	33.6	799	54.2	68055	6.7
2003	9602	6.8	1222	52.9	66781	−1.9
2004	12130	26.3	1894	55.0	67369	0.9
2005	14736	21.5	3182	68.0	66768	−0.9
2006	16784	13.9	4989	56.8	66490	−0.4
2007	17382	3.6	4765	−4.5	68281	2.7
2008	18962	9.1	5091	6.8	69997	2.5
2009	23613	24.5	5497	8.0	105640	50.9
2010	27360	15.9	7060	28.4	94609	−10.4

CT = computed tomography; MRI = magnetic resonance imaging; ED = Emergency Department.

**Figure 4:**
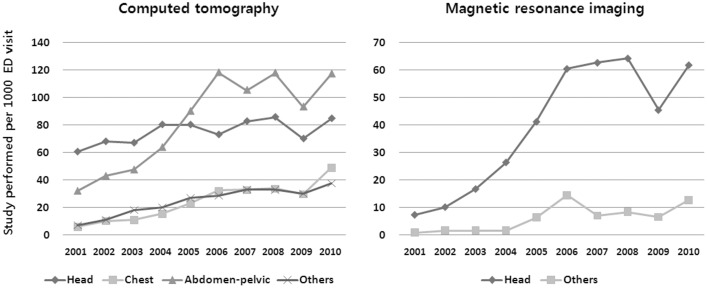
Anatomical regions and changes of studies performed by year. ED = Emergency Department.

The rates of CT use per 1000 ED visits increased across all body regions: head (ß = 2.0, P = 0.026); chest (ß = 4.3, P<0.001); abdomen-pelvic (ß = 9.9, P = 0.001); and others (ß = 3.2, P<0.001). The overall MRI utilization per 1000 ED visits also increased: head (ß = 6.7, P = 0.001); and others (ß = 1.2, P = 0.008). However, their relative increase throughout study period was particularly high for chest CT (eight-fold increase: 5.9 per 1000 ED visits in 2001 to 49.2 per 1000 in 2010) and head MRI (nine-fold increase: 7.2 per 1000 ED visits in 2001 to 61.9 per 1000 in 2010) (Figure 4).

## Discussion

We found a substantial overall increase in the use of CT and MRI in our ED between 2001 and 2010. The number of visits during which CT or MRI was performed increased by four-fold for CT scans and 13-fold for MRI. The data showed that older patients were more likely to undergo advanced radiology examination, i.e. CT and MRI, as part of their ED evaluation. This result was similar to that seen in previous studies. A US, multi-state analysis of the billing records from a national billing company showed that the CT utilization rate increased in each decade of life [2], and in a study using data from the National Hospital Ambulatory Medical Care Survey, CT use in the ED was greater in older patients than in younger ones [8].

Considering the number of annual ED patient visits, trends in the utilization rate of advanced radiology technology have also dramatically increased. Most of the figures in our study showing the trends of examinations performed have grooves in their curve for the year 2009. In 2009, as the number of patients visiting the ED increased due to the pandemic influenza A (H1N1) virus, this change reduced the rates of CT and MRI scans performed during that year. However, the total numbers of CT and MRI scan had constantly increased, as well as the overall utilization rate during the 10–year period. Although the rate of increase in the study utilization varies with the particular anatomical regions, most of the regions showed an increase much greater than the increase in patient volume over the same study period, and these trends occurred in both CT and MRI.

Various factors are considered to have contributed to the significant increase in the use of CT scanning in EDs. These include the increased availability of CT scanners [9], the proximity of CT scanners to EDs, the improved speed of new-generation scanners [10], the increased diagnostic indications of CT which have replaced conventional imaging modalities including CT angiography for aortic dissection, and non-contrast CT for the differential diagnose in renal colic, as well as the concerns regarding misdiagnosis or a decrease in clinical tolerance for diagnostic uncertainty [3]. Although the cost of MRI examinations is decreasing, thus making it more competitive with CT, there are not many common imaging scenarios in which MRI can simply replace CT scanning [10]. Nevertheless, the rate of increase in head MRI scans was far above that using head CT scans. Moreover, in certain conditions, a definite diagnosis can only be made by MRI. In the same context, diffusion-weighted brain MRI can be performed within a shorter examination time and it is widely used in various clinical situations in EDs [11]–[14], which may have influenced the dramatic increase in the use of head MRI scanning seen in our results.

A previous study reported that in the early 1990s the most common anatomical region examined by CT scanning was the head (55.4%), followed by the abdomen (14.2%), pelvis (12.0%), spine (11.5%), and chest (5.6%) [1]. Recent studies still show similar trends, except for a large increase in chest CT examinations as well as cervical spine CT [3], [15]. In our study, the overall utilization rate of chest CT increased by eight-fold during the study period. Because of the high absolute number of scans performed, the increase in the utilization of abdomen-pelvis CT was less dramatic; however, the absolute number of these scans still remains the highest. Although the absolute number of head scans remained the second largest among the CT study types, its increase rate was not significant. The nearly nine-fold increase in the rate of head MRI use might have contributed to blunting the increase in the number of head CT scans.

The increase in the number of advanced radiology examinations has also led to shortcomings, as increased cost is invariably associated with their use, CT scans provide a significant source of ionizing radiation exposure [15], there are physiological risks including contrast-induced nephropathy and allergic reactions [16], and an increased length of stay in the ED could lead to ED crowding and an increased risk of medical errors [17]. It is also unclear whether an increase in certain types of studies has improved the diagnostic yield [18]. The increase in the use of CT scanning may also fail to affect clinical outcomes [19]. A recent analysis of the 20-year trend of radiology utilization in a 793-bed, quaternary care, academic medical center showed increased imaging studies from 1993 to 2007, followed by a decline from 2007 through 2012, largely due to the decreased use of CT and MRI. The cause of this decline was mainly explained by the decrease of CT utilization after 2008, while the use of alternative imaging modalities with less ionizing radiation exposure increased [20]. The benefits and shortcomings of increased advanced radiology examination utilization in EDs have yet to be determined.

Our study has several limitations. First, as the data collected in this study could not represent EDs throughout Korea, our results may not be valid in other practice settings, such as rural or nonacademic urban centers. Second, we did not determine why there was radiology utilization for individual patients. There may be important considerations regarding the reasons for a particular study as the necessity and validity of workups in diseased vs. trauma patients are not the same. Something else to consider while interpreting the results, is the difference in the CT modality categorized in our study compared to previous ones. Some studies have defined ‘neck CT’ as a modality for evaluating the cervical spine [15], however, in our study, we classified this indication as ‘spine CT’, regardless of the anatomical regions, including the cervical, thoracic, lumbar, and sacral spine, and the ‘neck CT’ in our study represents CT scanning mainly used for the evaluation of soft tissue and the airway. Finally, additional information related to the benefits or harm of advanced radiology examinations would be informative, however, evaluating the outcome was beyond the scope of our study, and the analysis did not include patient outcomes following radiology utilization.

In this single center study, we reported a three-fold increase in the prevalence of CT usage and a nine-fold increase in the prevalence of MRI usage obtained during ED visits between 2001 and 2010. Although the increase occurred across a broad range of patients, the high prevalence of abdomen-pelvis CT and the dramatic increase in chest CT along with head MRI accounted for the majority of these changes, and we can provide a rationale regarding the increased need for resident training for interpreting these commonly performed radiologic examinations. Further studies are needed in order to understand the factors responsible for these changes and to determine the effect of advanced radiology utilization in EDs on the patient outcomes.
